# Tale of two nephropathies; co-occurring Alport syndrome and IgA nephropathy, a case report

**DOI:** 10.1186/s12882-021-02567-9

**Published:** 2021-10-30

**Authors:** Aniruddha Bhattacharyya, Yuting Huang, Sarah Hussain Khan, Cinthia Beskow Drachenberg, Laura C. Malone

**Affiliations:** 1 Department of Medicine, University of Maryland Medical Center Midtown Campus, Baltimore, MD 21201 USA; 2 Department of Medicine, University of Maryland School of Medicine, Baltimore, MD 21201 USA; 3 Department of Pathology, University of Maryland School of Medicine, 22 South Greene St., Baltimore, MD 21201 USA

**Keywords:** Alport syndrome, IgA nephropathy, Proteinuria, Hematuria, Case report

## Abstract

**Background:**

Alport Syndrome and IgA Nephropathy (IgAN) are both disorders that can cause hematuria. Alport syndrome is most commonly an X-linked disease, caused by COL4A5 mutation. Mutations of COL4A3 and COL4A4 on chromosome two are also common causes of Alport syndrome. IgAN is the most common glomerulonephritis worldwide. Though IgAN is usually sporadic, an estimated 15% of cases have an inheritable component. These cases of Familal IgA Nephropathy (FIgAN) can have mutations on genes which are known to cause Alport Syndrome.

**Case presentation:**

We report a case of a 27-year-old man with strong family history of renal disease, who presented with hematuria and new non-nephrotic range proteinuria. Physical exam showed no abnormalities. His creatinine remained persistently elevated, and renal ultrasound exhibited bilaterally increased echogenicity consistent with Chronic Kidney Disease. Twenty-four-hour urinary collection revealed non-nephrotic range proteinuria of 1.4 g, with otherwise negative workup. On biopsy, he had IgA positive immunofluorescent staining as well as moderate interstitial fibrosis and tubular atrophy. Electron microscopy showed a basket-weave pattern of thickening and splitting of the lamina densa-consistent with Alport Syndrome, as well as mesangial expansion with electron-dense deposits -consistent with IgAN.

**Conclusions:**

Mutations of COL4A5 on the X chromosome, as well as mutations of COL4A3 and COL4A4 on chromosome 2, can cause both Alport Syndrome and FIgAN. Genome wide association studies identified certain Angiotensin Converting Enzyme gene polymorphisms as independent risk factors for progression of IgAN. Our Presentation with this co-occurring pathology suggests a new paradigm where Alport Syndrome and FIgAN may represent manifestations of a single disease spectrum rather than two disparate pathologies. Appreciating hematuria through this framework has implications for treatments and genetic counseling. Further genome wide association studies will likely increase our understanding of Alport Syndrome, FIgAN, and other causes of hematuria.

## Background

Alport Syndrome and IgA Nephropathy (IgAN) are both causes of painless hematuria and proteinuria. IgAN is the most common glomerulonephritis worldwide, and usually occurs sporadically, though familial inheritance of IgAN has been described [1, 2]. Alport Syndrome is a rare familial disorder of type IV collagen occurring in approximately 1 in 50,000 live births [3, 4], and is most often caused by X-linked mutation in the COL4A5 gene. History and acuity of presentation of these diseases are often distinct, but they can overlap. Definitive diagnosis is made from histologic findings seen on kidney biopsy.

It can be difficult to distinguish familial IgA nephropathy (FIgAN) from Alport Syndrome based on clinical history [5, 6]. Various genes that have been implicated in FIgAN can overlap with the causative genes of Alport Syndrome [5–8]. Here we investigate a patient with hematuria and new-onset kidney disease, who had co-occurring Alport Syndrome and IgAN.

## Case presentation

A 27-year-old male with no prior past medical history presented to the Emergency Department complaining of 3 days of increased urinary frequency, dysuria, nocturia, and intermittent hematuria. The patient indicated episodes of tinnitus that predated urinary symptoms. He reported no constitutional symptoms and denied noticeable hearing loss, visual changes, flank pain, polydipsia, penile discharge, or genital sores prior to presentation. Notably, multiple family members had a history of kidney disease. His mother has chronic kidney disease (CKD) with no pertinent histologic findings on renal biopsy. The patients’ elder brother died from renal failure of unknown cause at age 26 years.

Our patient presented with a blood pressure of 160/85 mmHg. His vitals were otherwise within normal limits. Physical exam showed no abdominal or costovertebral angle tenderness; his cardiopulmonary, ophthalmologic, and lower extremity exams showed no abnormalities. His labs were notable for elevated creatinine of 176.8 μmol/L (2.0 mg/dL), with a BUN of 5.7 mmol/L (16 mg/dL). Urine Dipstick revealed the presence of 2+ blood, 3+ protein; urine microscopy showed 6–10 Red Blood Cells (RBC) per high-powered field. His fractional excretion of sodium (FENa) was 0.6%, and serum osmolality was 295 mmol/kg (295 mOsm/kg).

The patient was admitted for observation due to his elevated blood pressure and pre-renal acute kidney injury with proteinuria and microscopic hematuria. He received intravenous fluids, however, creatinine remained between 165.35 to 171.53 μmol/L (1.87 to 1.94 mg/dL). The calculated GFR was 48 mL/min/1.73 m^2^ using the CKD-EPI Creatinine equation. Twenty-four-hour urinary collection revealed non-nephrotic range proteinuria of 1.4 g. Further workup during this hospitalization, including serum creatinine kinase, protein electrophoresis, C3, C4, antinuclear antibodies (ANA), and antineutrophil cytoplasmic antibodies (ANCA), were all within normal limits. Renal ultrasound showed bilaterally increased echogenicity consistent with CKD, and no calculi or signs of hydronephrosis.

**Fig. 1:**
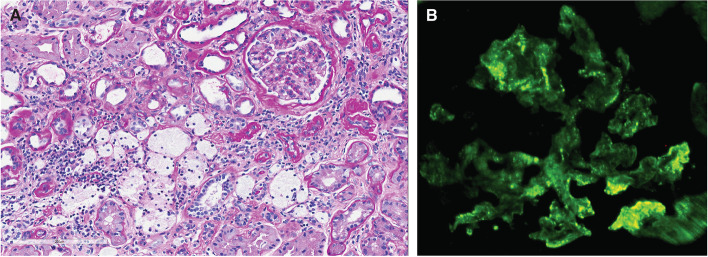
**A** PAS stain showing glomerulus with mild increase in mesangial matrix and cellularity consistent with IgA nephropathy and adjacent foam cells within the interstitium, which are frequently seen in but not specific to Alport Syndrome. **B** Immunofluorescent stain positive for granular IgA deposits (3+) within the mesangium consistent with IgA nephropathy

Subsequently, the patient underwent kidney biopsy for workup of his CKD and hematuria. Light microscopy revealed moderate interstitial fibrosis with tubular atrophy and multifocal scarring involving 40–45% of the sampled cortical areas. The glomeruli showed an increase in mesangial cellularity and a mild increase in mesangial matrix with no clear endocapillary proliferation, segmental sclerosis, or crescents. There were also abundant lipid laden foam cells present within the interstitium (Fig. 1A).

**Fig. 2:**
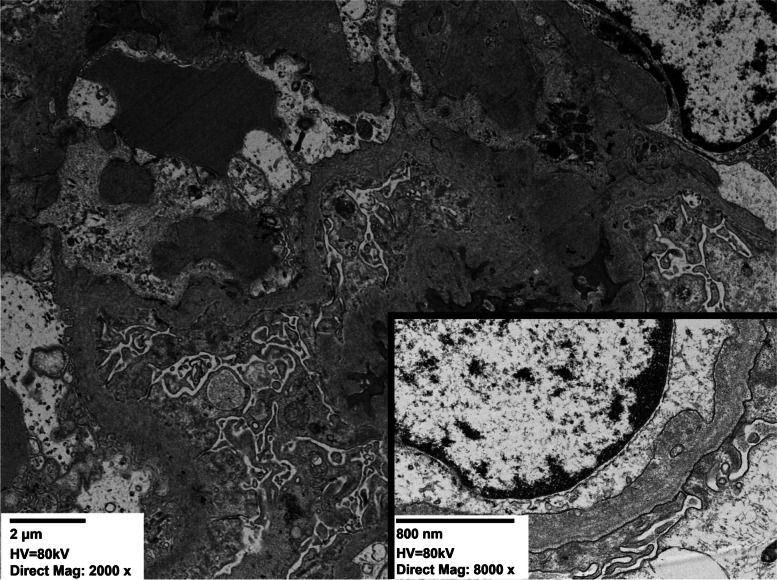
Electron Micrograph showing mesangial expansion with matrix hypercellularity, and unevenly distributed globular electron-dense immune-type deposits consistent with IgA. Top Left Inset shows high magnification of GBM, which exhibits thickening and splitting of the lamina densa with a basket-weave appearance, as well as electron-dense granules within the lucent zones between layers

Immunofluorescent staining was positive for IgA (3+) and C3 (2+) showing a granular pattern of deposition in the mesangium (Fig. 1B). Immunofluorescent stains for IgG, IgM, C1q, albumin, and fibrinogen were negative. Electron microscopy showed mesangial expansion with electron-dense deposits consistent with IgAN [1, 2]. However, the glomerular basement membrane (GBM) exhibited irregular thickening and splitting of the lamina densa with a basket-weave appearance, and heterogeneous electron-lucent areas which are a typical characteristic appearance of the GBM in Alport Syndrome (Fig. 2) [3, 4], and are not seen in IgAN.

Based on the combined findings seen on immunofluorescence and electron microscopy, the patient was diagnosed with both Alport Syndrome and IgAN. He did not have any genetic testing or audiologic testing performed during the workup and management of these disorders. During admission, amlodipine was started to treat hypertension but was later switched to lisinopril to manage hypertension and proteinuria. As his 24-h urine protein collection showed non-nephritic range proteinuria of 1.4 g, further steroids or immunosuppression was not required in this case.

## Discussion and conclusions

Alport Syndrome and IgAN are both renal disorders that can present with hematuria [1–4]. Distinguishing these diseases is based primarily on family history and characteristic histological findings [6]. Treatment for IgAN involves no treatment for hematuria with minimal proteinuria, angiotensin converting enzyme (ACE) inhibitors for proteinuria over 0.5 g in 24 h, and steroids with or without immunosuppression for nephrotic range proteinuria or cresentic IgAN seen on histology [1, 2]. There is no specific treatment for Alport syndrome, though observational studies suggest ACE inhibitors may delay the onset of renal failure [3, 4]. This patient was continued on the ACE inhibitor lisinopril, for concurrent management of his IgAN with non-nephrotic proteinuria of 1.4 g over 24 h, his Alport syndrome, and hypertension.

Alport Syndrome is most commonly due to the X-linked inheritance of COL4A5 mutations (85% of cases), though autosomal recessive inheritance of COL4A3 and COL4A4 gene mutations often occur [3, 4]. For patients with Alport Syndrome, age of symptom onset and progression to End Stage Renal Disease (ESRD) is consistent amongst family members [3, 4]. For X-linked Alport Syndrome, the risk of progression to ESRD before age 30 is 90% [3, 4].

For IgAN, the pathologic features seen on kidney biopsy are used to prognosticate disease progression based on the revised Oxford Classification system [1, 2]. The degree of tubular atrophy or interstitial fibrosis is the strongest predictive factor for the progression of renal disease. While the majority of IgAN cases are sporadic, an estimated 15% have a heritable component [9]. Genome wide association studies (GWAS) identified certain ACE gene polymorphisms as independent risk factors for disease progression [10] and implicated multiple genes in the pathogenesis of FIgAN, including mutations occurring in the genes that also cause Alport Syndrome [5–9]. A disease susceptibility locus for FIgAN has been identified at locus 2q36 [5]. This region includes the two adjacent genes COL4A3 and COL4A4 (2q36.3) which can mutate to cause Alport syndrome [11]. Additionally, a likely pathologic variant of COL4A5 (Xq22.3) was detected in one family with FIgAN [7]. Mutations in this gene are the most common cause of Alport Syndrome [3, 4].

Our patient’s histologic findings were consistent with both Alport Syndrome and IgAN. In the context of family history and known genetic overlap between these renal diseases, this case suggests a new paradigm where Alport Syndrome and FIgAN may represent manifestations of a single disease spectrum rather than two disparate pathologies. Viewing hematuria through this paradigm has potential testing, counseling, and treatment implications.

Genetic testing may be warranted in cases of IgAN when accompanied by risk factors of a familial pattern; ie. family history of renal disease, or the presence of histologic features suggesting Alport Syndrome. This identifies a clade of patients with a potential familial component who would benefit from early genetic counseling. Currently, genomic testing can locate multiple single nucleotide polymorphisms (SNP) in genes implicated in IgAN via complementary oligonucleotide hybridization with nucleotides of target genes harboring mutations [12]. Patients with IgAN and genetic test results showing mutations in genes known to cause Alport Syndrome may benefit from the earlier introduction of ACE inhibitors before increased proteinuria occurs.

As a corollary, genetic testing could also be used in patients with Alport Syndrome to determine if they have additional mutations associated with FIgAN [12].. Further, genetic testing in patients with Alport syndrome also can help determine the need for steroid-based therapies. Data would be needed to determine if the addition of steroids and immunomodulation would benefit patients with Alport Syndrome who have genetic profiles similar to FIgAN. Genomic testing can also be used to locate SNPs on genes which appear to reduce the risk of developing IgAN [12], though further studies would be required to assess prognosticating value for patients with Alport Syndrome. Finally, we suggest the adoption of widespread genetic screening, allowing for more retrospective and prospective GWAS [9]. Such studies have the potential to increase our understanding of Alport Syndrome and FIgAN as well as other potential causes of hematuria.

## Data Availability

Data sharing is not applicable to this article as no datasets were generated or analysed during the current study.

## Abbreviations

IgAN: IgA Nephropathy
FIgAN: Familial IgA Nephropathy
CKD: Chronic Kidney Disease
ANA: Antinuclear Antibodies
ANCA: Antineutrophil Cytoplasmic Antibodies
GBM: Glomerular Basement Membrane
ACE: Angiotensin Converting Enzyme
ESRD: End Stage Renal Disease
GWAS: Genome Wide Association Studies
SNP: Single Nucleotide Polymorphism.
